# Measurement for the Area of Red Blood Cells From Microscopic Images Based on Image Processing Technology and Its Applications in Aplastic Anemia, Megaloblastic Anemia, and Myelodysplastic Syndrome

**DOI:** 10.3389/fmed.2021.796920

**Published:** 2022-01-25

**Authors:** Yongfeng Zhao, Tingting Huang, Xian Wang, Qianjun Chen, Hui Shen, Bei Xiong

**Affiliations:** ^1^Department of Hematology, Zhongnan Hospital of Wuhan University, Wuhan, China; ^2^Department of Hematology, The First Affiliated Hospital of Yangtze University, Jingzhou, China; ^3^Department of Pharmacy, The First Affiliated Hospital of Yangtze University, Jingzhou, China; ^4^National Engineering Research Center for E-Learning, Central China Normal University, Wuhan, China; ^5^The State Key Laboratory of Biocatalysis and Enzyme Engineering of China, College of Life Sciences, Hubei University, Wuhan, China

**Keywords:** myelodysplastic syndrome, megaloblastic anemia, aplastic anemia, image processing technology, area of red blood cells

## Abstract

**Background:**

Aplastic anemia (AA), megaloblastic anemia (MA), and myelodysplastic syndrome (MDS) were common anemic diseases. Sometimes it was difficult to distinguish patients with these diseases.

**Methods:**

In this article, we proposed one measurement method for the area of red blood cells (RBCs) from microscopic images based on image processing technology and analyzed the differences of the area in 25 patients with AA, 64 patients with MA, and 68 patients with MDS.

**Results:**

The area of RBCs was 44.19 ± 3.88, 42.09 ± 5.35, 52.87 ± 7.68, and 45.75 ± 8.07 μm^2^ in normal subjects, patients with AA, MA, and MDS, respectively. The coefficients of variation were 8.78%, 10.05%, 14.53%, and 14.00%, respectively, in these groups. The area of RBCs in patients with MA was significantly higher than normal subjects (*p* < 0.001). Compared with patients with AA and MDS, the area of RBCs in patients with MA was also significantly higher (*p* < 0.001). The results of correlation analysis between the area of RBCs and mean corpuscular volume (MCV), mean corpuscular hemoglobin (MCH), MCH concentration (MCHC), and red cell distribution width showed no significant correlations (*p* > 0.05). The area under the curve (AUC) results of the Receiver Operating Characteristic (ROC) curves of RBCs area were 0.421, 0.580, and 0.850, respectively, in patients with AA (*p* = 0.337), MDS (*p* = 0.237), and MA (*p* < 0.001).

**Conclusion:**

Identifying the area of RBCs in peripheral blood smears based on the image processing technology could achieve rapid and efficient diagnostic support for patients with MDS and MA, especially for patients with MA and in combination with MCV. However, a larger sample study is needed to find the cutoff area values.

## Introduction

Myelodysplastic syndrome (MDS) is a group of heterogeneous clonal diseases originated from hematopoietic stem cells, which is characterized by ineffective hematopoiesis, refractory hemocytopenia, and high-risk transformation to acute myeloid leukemia (1). Aplastic anemia (AA) is a group of diseases that result in the decrease of blood cells due to acquired bone marrow failure (2, 3). Megaloblastic anemia (MA) is an anemic disease caused by the disorder of DNA synthesis of blood cells, which is characterized by the megaloblastic metamorphosis of red blood cells (RBCs) and myeloid cells. Vitamin B12 and/or folate deficiency are the most common causes of MA (4). It is sometimes difficult to make differential diagnoses among these three diseases according to clinical manifestations and blood examinations because of similar findings shared by them. Anemia, bleeding, and infections due to cytopenia in one or more lineages can be seen in all these diseases mentioned above. Dysplasia in lineages of peripheral blood was shown not only in MDS, but also in MA. Especially, megaloblastic metamorphosis of erythrocytes in myelogram of patients with MDS needs to be differentiated with MA. Some patients with MDS who do not display prominent dysplasia are difficult to be made differential diagnoses with patients with AA. Sometimes, it was difficult to distinguish patients with MA from MDS of refractory anemia (RA) type (MDS-RA) (5) and multilineage dysplasia type (MDS-MLD), as well as difficult to distinguish patients with AA from hypoplastic MDS. The detection of folate and vitamin B12 is helpful to diagnose MA. Finding the clonal evidence of MDS is helpful to diagnose MDS. There were possibly 52% of patients who had one or more clonal chromosome abnormalities (6). The acquired molecular mutations were possibly found in 80–90% of patients with MDS (7, 8). However, the limitations of testing cost and laboratory test conditions in many low-resource settings make detections of abnormal chromosomes, molecular mutations, and concentrations of vitamin B12 and folate not available for some patients suspected of MDS, AA, and MA.

In this study, we described a measurement method based on image processing technology, which was developed to localize and extract RBCs from microscopic images and further calculated the area of RBCs for the first time. It can help for the diagnosis and identification of MDS, AA, and MA because the hematological analyzer can release the result within minutes.

## Methods

### Patients and Diagnosis

Myelodysplastic syndrome was diagnosed according to the 2016 WHO classification (9). The diagnosis of AA was made according to the standard published by the British Society for Standards in Haematology in 2016 (10). Patients with vitamin B12 values below 150 pmol/l were diagnosed with MA. This study and all the procedures used were approved by the Institutional Review Board of the Wuhan University, China. All the patients were from Zhongnan Hospital of Wuhan University. There were 25 normal subjects, 25 patients with AA, 64 patients with MA, and 68 patients with MDS.

### Measurement Method

**Figure 1:**
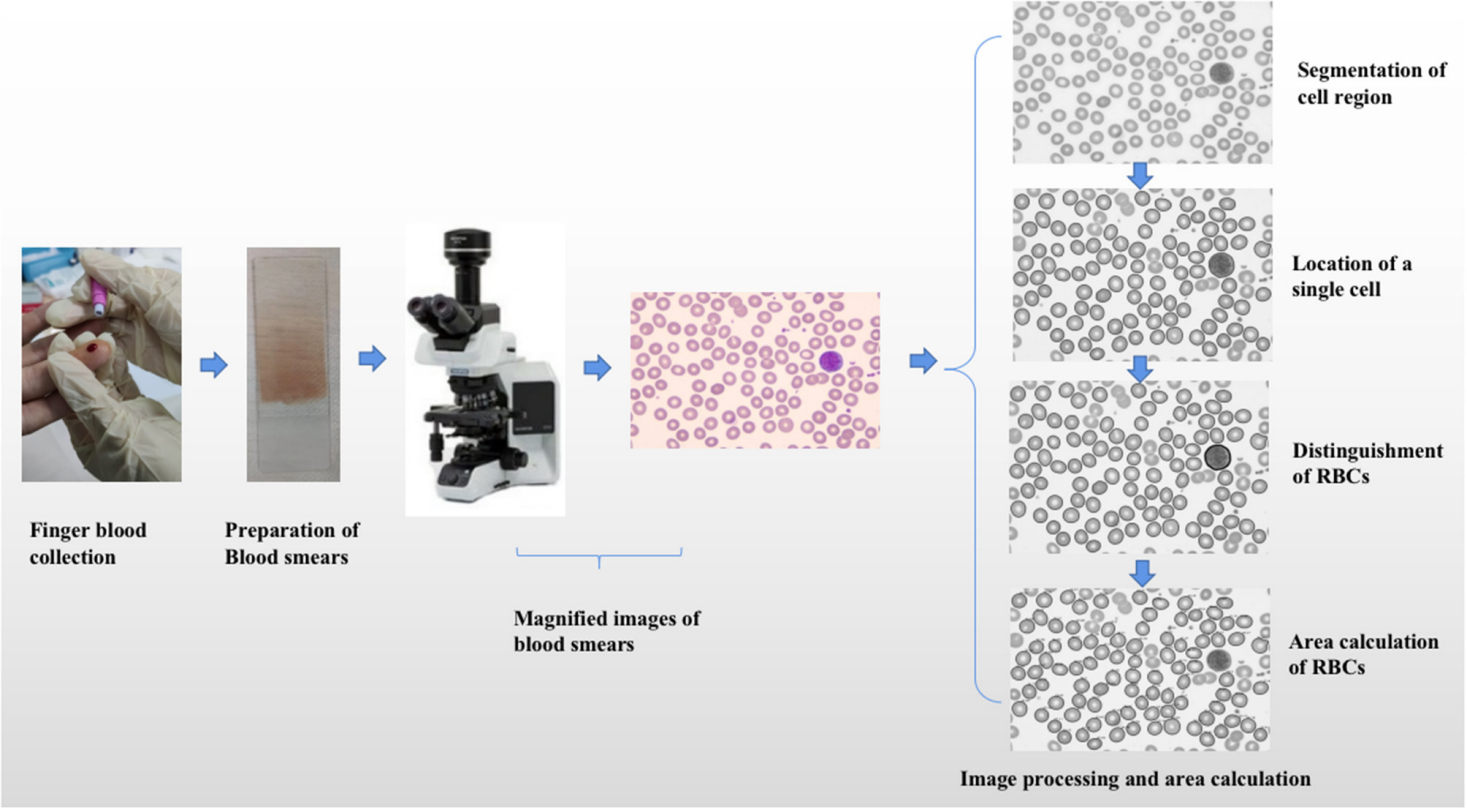
The specific process of area calculation of red blood cells. The specific process included preparation of blood smears, magnified images of blood smears, and area calculation of red blood cells.

We used a machine learning method based on image processing technology to obtain the area of RBCs in normal subjects or patients with anemia. The specific process included preparation of blood smears, magnified images of blood smears, and area calculation of RBCs (shown in Figure 1).

### Preparation of Blood Smears

Blood smears at the time of initial diagnosis or entering the study were acquired from all patients or subjects. The following steps were observed to obtain blood smears. At first, 5–7 μl of Ethylene Diamine Tetraacetic Acid (EDTA) anticoagulated peripheral blood or one drop of peripheral blood was directly collected from all subjects and patients, and the blood collected was dripped to 1 cm at one end of the slide or 3/4 end of the whole slide. Then we pushed the cover slide close to the blood drop, gently touched the blood drop, pressed it on the blood drop, and filled the width of the slide. Finally, Wright-Giemsa mixed staining was needed.

### Magnified Images of Blood Smears

The magnified images of peripheral blood smears were obtained by microscope (magnified 1,000 times). The obtained magnified images were input into the computer system, which were Red Green Blue (RGB) images or hue, saturation, and value Hue Saturation Value (HSV) images.

### Area Calculation of RBCs

#### Segmentation of Cell Region and Background

The first step was converting the RGB space to HSV space about images, which included HSV. Hue contains hue and hue information. Saturation contains saturation and color purity information. Value contains lightness information. The next step was image segmentation, which included image threshold segmentation and edge-based segmentation. Otsu threshold segmentation method was mainly used for image segmentation.

#### Location of the Single Cell in the Image

The outer contour of the image was extracted in the cell region by the findCounter method. After traversing all outer contours, the pending contour was the outer contour with an area less than the threshold (0.5–10 μm^2^). *S*_*mean*_ was the average area of all outer contours except the pending contour.

The convex hull algorithm was used to identify the effective contour. The cell with the contour of which was convex edge shape was selected as a single cell. After traversing all outer contours except the pending contour, the outer contour with the area of which was in the specific range (*a* × *S*_*mean*_, *b* × *S*_*mean*_) was the effective contour. The value of a was less than 1, and the value of b was greater than 1. After many tests, the accuracy was best when a was 0.3 and b was 5. When the area of one outer contour was larger than *b* × *S*_*mean*_, it would be judged as multiple cells merging.

#### Distinguishment of RBCs From Nucleated Cells

Single cells include nucleated cells and RBCs. After the above location of single cells, they also should be divided into nucleated cells and RBCs. The mean gray value of all located single cells images was calculated and recorded as Mg. The single gray value of each single cell image was also calculated and recorded as G. If the ratio of G to Mg was greater than 0.8, the single cell would be selected as a RBC. If the ratio of G to Mg was less than 0.8, the single cell would be selected as a nucleated cell.

#### Area Calculation of RBCs

If the ratio of the distance from all points to the center of contour (r_i_) to the radius (r) of the minimum enclosing circle was above 0.85, and the area of the minimum enclosing circle was in the special range (*a* × *S*_*mean*_, *b* × *S*_*mean*_), the cell would be labeled as a recall RBC. The average ratio of r_i_ to r was named as *P*_*mean*_.

The area of the selected RBC was labeled as S_i_, the area of recall RBC was labeled as S_r._ The area of recall RBC was calculated based on the idea of calculus and according to the following formula. Finally, we calculated the average area of all RBCs in the blood smear in every subject and patient.


$$\begin{array}{l} S_{\text{i}}=\pi \times r\times r\\ S_{\text{r}}=Si\;\times P_{mean}\times P_{mean} \end{array}$$

### Statistical Analysis

The normality of area values was carried by the Shapiro–Wilk test. The area was expressed by “mean ± SD” or “median ± quartile range.” If the area values satisfied normal distribution and homogeneous variance, they would be analyzed by an independent sample *t*-test. If the distribution was normal but the variance was not uniform, the corrected *t*-test would be used. If the normal distribution could not be satisfied, the rank-sum test would be used for analysis. The coefficient of variation was calculated by the ratio of mean to standard deviation. Pearson or Spearman correlation analysis between the area and standard RBC complete blood count (CBC) indices were used according to the normality of data. The ROC curves and the values of the area under the curve (AUC) were used to compare the area of RBCs with MCV.

## Results

### Characteristics of Normal Subjects and Patients With AA, MDS, and MA

In all the normal subjects, the average number of RBCs studied was 86.80. In patients with AA, MDS, and MA, the average numbers were 88.84, 113.97, and 106.73, respectively. Table 1 shows the characteristics of normal subjects and all the patients included in this study. The average or median values of mean corpuscular volume (MCV) in patients with AA, MDS, and MA were 95.82, 100.80, and 123.26 fl, respectively. The average or median values of mean corpuscular hemoglobin (MCH) in patients with AA, MDS, and MA were 33.22, 33.21, and 41.91 pg, respectively. The average or median values of MCH concentration (MCHC) and red cell distribution width (RDW) were seen in Table 1.

**Table 1:** The characteristics of normal subjects and patients with AA, MDS, and MA.

	**Normal subjects**	**AA**	**MDS**	**MA**
Sex [(male,%)]	14(56%)	17(68%)	30(44%)	37(58%)
Age (years)	32.88 ± 9.13	47.36 ± 21.33	63.87 ± 13.51	60.73 ± 7.62
WBC (109/L)	5.88 ± 1.42	1.76 ± 0.65	2.62 ± 2.67	3.77 ± 3.37
Hemoglobin (g/L)	129.49 ± 9.58	66.29 ± 14.21	63.10 ± 17.53	61.30 ± 14.57
RBC (10^12^/L)	4.80 ± 0.69	2.21 ± 0.65	2.49 ± 1.05	2.01 ± 0.98
PLT (10^9^/L)	241.24 ± 58.30	33.64 ± 12.95	58.50 ± 65.50	64.50 ± 37.50
MCV(fL)	89.94 ± 5.87	95.82 ± 14.25	100.80 ± 13.33	123.26 ± 13.28
MCH(pg)	29.60 ± 2.33	33.22 ± 5.12	33.21 ± 4.92	41.91 ± 4.93
MCHC (g/L)	328.44 ± 9.93	347.05 ± 15.01	328.90 ± 14.59	340.84 ± 11.47
RDW(%)	13.76 ± 1.66	15.20 ± 3.91	18.90 ± 5.45	19.16 ± 4.84

*WBC, white blood cell; RBC, red blood cell; PLT, platelet; MCV, mean corpuscular volume; MCH, mean corpuscular hemoglobin; MCHC, mean corpuscular hemoglobin concentration; RDW, red cell distribution width; AA, aplastic anemia; MA, megaloblastic anemia; MDS, myelodysplastic syndrome*.

### Area of RBCs in Normal Subjects and Patients With AA, MDS, and MA

**Figure 2:**
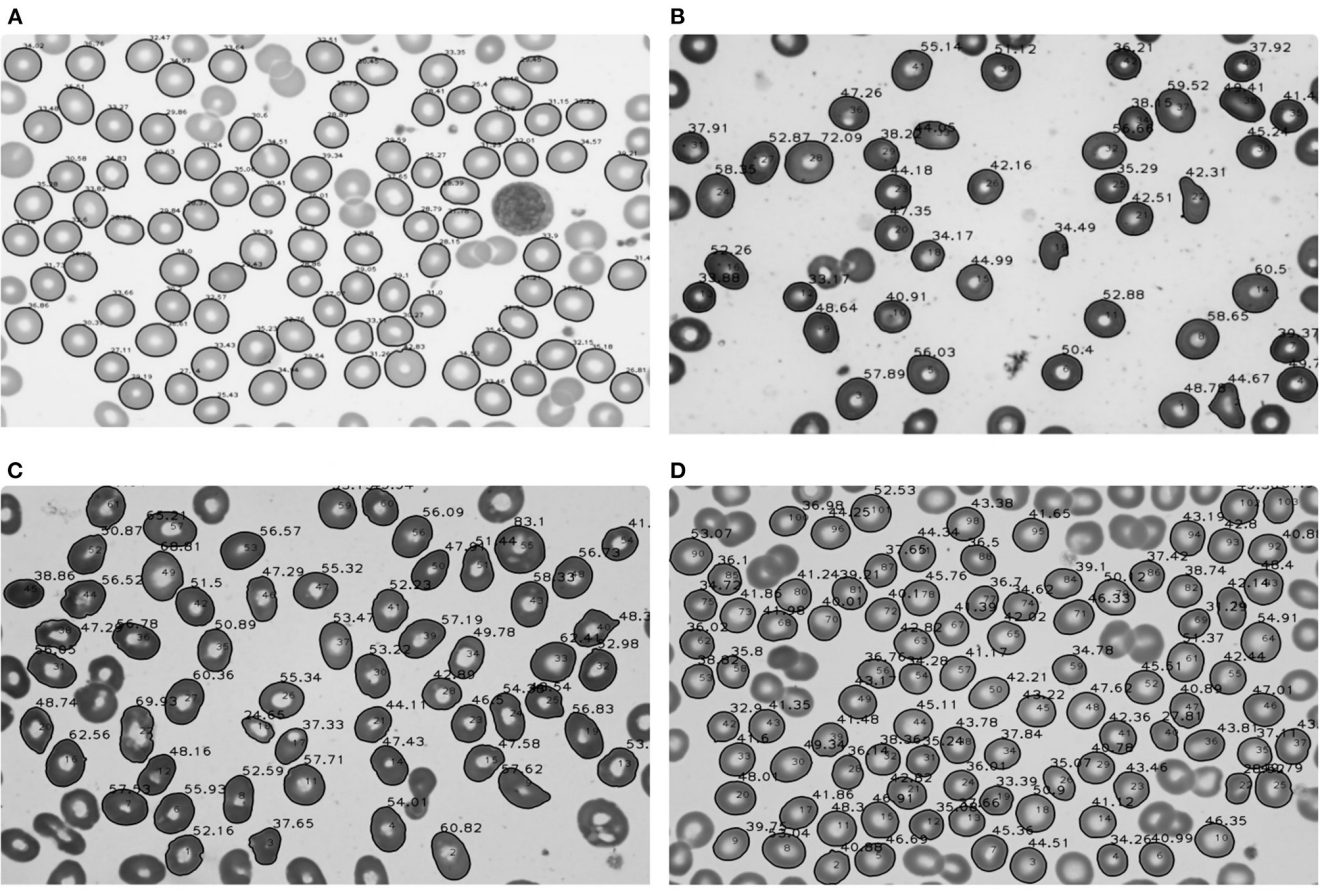
Area calculation results of red blood cells in normal subjects and patients with aplastic anemia (AA), megaloblastic anemia (MA), and myelodysplastic syndrome (MDS). **(A)** Area calculation results in one normal subject; **(B)** Area calculation results in one patient with AA; **(C)** Area calculation results in one patient with MA; **(D)** Area calculation results in one patient with MDS.

**Figure 3:**
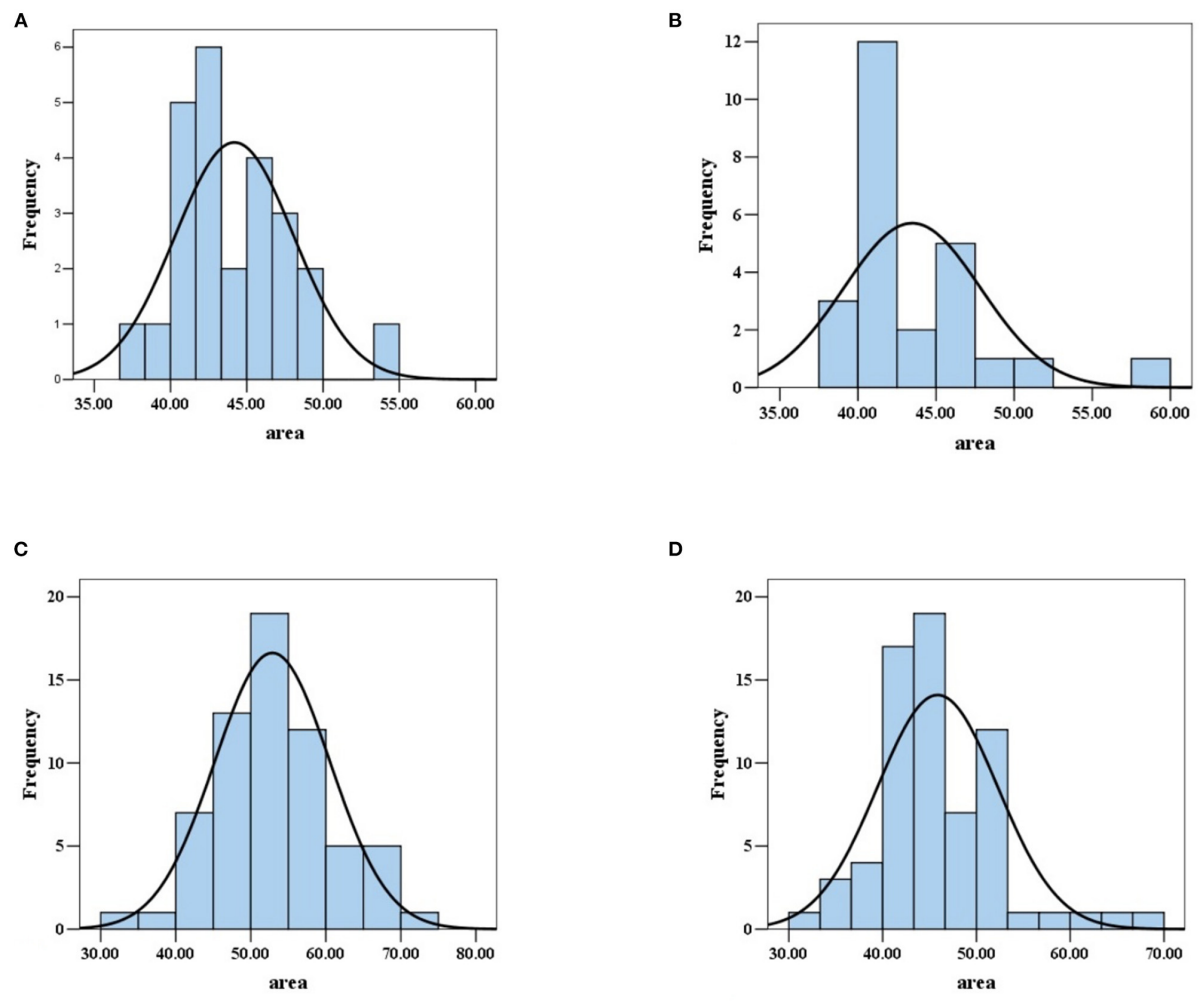
Area values distribution of red blood cells in normal subjects and patients with AA, MA, and MDS. **(A)** The area values of red blood cells in normal subjects satisfied normal distribution. **(B)** The area values of red blood cells in patients with AA did not satisfy normal distribution. **(C)** The area values of red blood cells in patients with MA satisfied normal distribution. **(D)** The area values of red blood cells in MDS patients did not satisfy normal distribution.

In 25 normal subjects, the mean area was 44.19 μm^2^, and the standard deviation was 3.88 μm^2^ (Table 2). The image result of the area calculation in one normal subject is shown in Figure 2A. The area values of RBCs in normal subjects satisfied normal distribution (Figure 3A). The coefficients of variation (CV) were 8.78%.

**Table 2:** The results of area in normal subjects and patients with AA, MA, and MDS.

	**Mean**	**Median**	**Standard deviation**	**Quartile range**	***P*-value**
	**(μm^**2**^)**	**(μm^**2**^)**	**(μm^**2**^)**	**(μm^**2**^)**	**(Shapiro-Wilk)**
Normal subjects	44.19	43.01	3.88	5.47	0.561
AA patients	43.47	42.09	4.37	5.35	0.003
MA patients	52.87	51.72	7.68	10.84	0.637
MDS patients	45.86	45.75	6.42	8.07	0.006

*AA, aplastic anemia; MA, megaloblastic anemia; MDS, myelodysplastic syndrome*.

In 25 patients with AA, the median area was 42.09 μm^2^, the quartile range was 5.35 μm^2^. In 68 patients with MDS, the median area was 45.75 μm^2^, the quartile range was 8.07 μm^2^. In 64 patients with MA, the mean area was 52.87 μm^2^, and the standard deviation was 7.68 μm^2^ (Table 2). The image results of area calculation in one patient with AA, MA, and MDS are, respectively, shown in Figures 2B–D. The area values of RBCs in MA patients satisfied normal distribution, not patients with AA and MDS (Figures 3B–D). The CV were 10.05, 14.53, and 14.00%, respectively in patients with AA, MA, and MDS.

### Differences Between the Area of Normal Subjects and Patients With AA, MDS, and MA

Compared with normal subjects, the area of RBCs in patients with MA showed significant differences (52.87 ± 7.68 vs. 44.19 ± 3.88 μm^2^, *p* < 0.001). The area of patients with AA and MDS showed no significant differences compared with normal subjects (42.09 ± 5.35 vs. 44.19 ± 3.88 μm^2^, *p* = 0.337) (45.75 ± 8.07 vs. 44.19 ± 3.88 μm^2^, *p* = 0.237).

### Differences Between the Area of Patients With AA, MDS, and MA

Compared with the area of RBCs in patients with MA, patients with MDS and AA showed significantly smaller area of RBCs, respectively (45.75 ± 8.07 vs. 52.87 ± 7.68 μm^2^, *p* < 0.001) (42.09±5.35 vs. 52.87±7.68 μm^2^, *p* < 0.001). There were also significant differences between patients with MDS and AA (45.75 ± 8.07 vs. 42.09 ± 5.35 μm^2^, *p* = 0.048).

### Correlation Analysis Between the Area of RBCs and Standard Red Blood Cell CBC Indices

In all patients with AA, the results of correlation analysis between the area **of** RBCs and MCV showed no significant correlation (*r* = −0.173, *p* = 0.409). No significant correlations were also showed between area and MCH (*r* = 0.065, *p* = 0.758), area and MCHC (*r* = 0.244, *P* = 0.240), area and RDW (*r* = −0.239, *p* = 0.250). In all patients with MDS, the correlation analysis between the area **of** RBCs and MCV also showed no significant correlation (*r* = −0.038, *p* = 0.760). No significant correlations were also showed between area and MCH (*r* = −0.010, *p* = 0.934), area and MCHC (*r* = 0.066, *p* = 0.595), area and RDW (*r* = −0.085, *p* = 0.490). In all patients with MA, the results of correlation analysis between the area of RBCs and MCV (*r* = −0.039, *p* = 0.760), MCH (*r* = −0.015, *p* = 0.906), MCHC (*r* = −0.030, *p* = 0.812), RDW (*r* = −0.049, *p* = 0.698) showed no significant correlations (Table 3).

**Table 3:** Correlation analysis between the area of RBCs and standard red blood cell indices.

	**r^a^**	**r^b^**	**r^c^**	**r^d^**	***P* ^a^**	***P* ^b^**	***P* ^c^**	***P* ^d^**
Normal subjects	0.02	−0.113	−0.275	0.335	0.924	0.592	0.184	0.102
AA	−0.173	0.065	0.244	−0.239	0.409	0.758	0.240	0.250
MDS	−0.038	−0.01	0.066	−0.085	0.760	0.934	0.595	0.490
MA	−0.039	−0.015	−0.03	−0.049	0.760	0.906	0.812	0.698

a *for MCV;*

b *for MCH;*

c *for MCHC;*

d *for RDW*. *AA, aplastic anemia; MA, megaloblastic anemia; MDS, myelodysplastic syndrome; RBCs, red blood cells*.

### Comparison of the Area of RBCs With MCV

**Figure 4:**
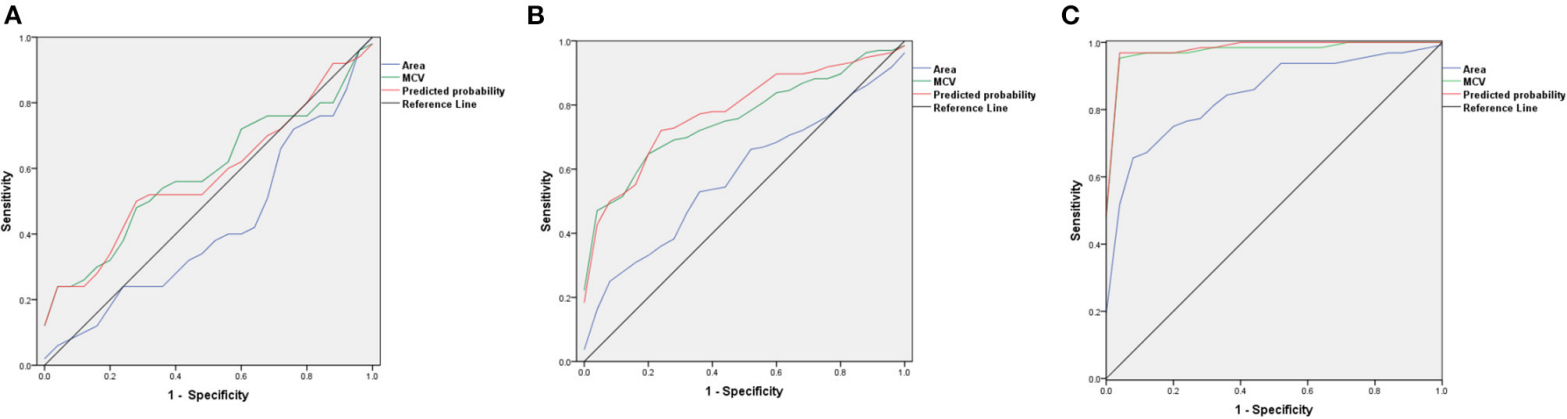
The ROC curves of red blood cells (RBCs) area, mean corpuscular volume (MCV), and predicted probability in patients with AA, MDS, and MA. **(A)** The ROC curves in patients with AA; **(B)** The ROC curves in patients with MDS; **(C)** The ROC curves in patients with MA.

We compared the AUC results of ROC curves of RBCs area, MCV, and predicted probability of the two indicators in patients with AA, MA, and MDS. In patients with AA, the AUC results of RBCs area, MCV, and predicted probability were 0.421, 0.585, and 0.581, respectively. We found that MCV and RBCs area had little diagnostic significance in patients with AA. In patients with MDS, the AUC results of RBCs area, MCV, and predicted probability were 0.580, 0.763, and 0.784, respectively. The area of RBCs did not show obvious advantages, but the diagnostic value of the combination of the two indexes increased (*p* = 0.048). In patients with MA, the AUC results of RBCs area, MCV, and predicted probability were 0.850, 0.984, and 0.991, respectively. The area of RBCs showed a very good diagnostic value and the combined diagnostic value of the two indexes increased significantly (*p* < 0.001). These results are shown in Figure 4 and Table 4.

**Table 4:** The AUC results of RBCs area, MCV, and predicted probability in patients with AA, MA, and MDS.

	**AA**		**MDS**		**MA**	
	**AUC**	***P***	**AUC**	***P***	**AUC**	***P***
Area	0.421	0.337	0.580	0.237	0.850	0.000
MCV	0.586	0.295	0.763	0.000	0.984	0.000
Predicted probability	0.581	0.327	0.784	0.000	0.991	0.000

*AA, aplastic anemia; MA, megaloblastic anemia; MDS, myelodysplastic syndrome; AUC, area under the curve; MCV, mean corpuscular volume*.

## Discussion

Myelodysplastic syndrome, AA, and MA are three common hematologic diseases that cause pancytopenia. MDS and MA possibly share similar characteristics in bone marrow cells morphology, such as abnormal nuclear division and nuclear morphology of erythrocyte lines, which make doctors difficult to distinguish them. The diagnostic distinction of AA and hypocellular MDS is also difficult because of some shared clinical features such as bone marrow hypocellularity (11). Dysplasia in one or more hematopoietic cell lineages is a prerequisite for the diagnosis of MDS. However, dysplasia is not specific for MDS. A small number of patients with MDS display no dysplasia in the early stage of the disease (12, 13). The proper diagnostic distinction of these diseases with pancytopenia is challenging.

In bone marrow morphology, the number of blasts, pseudo-Pelger–Huet anomaly, and micro megakaryocytes are of great diagnostic value for MDS (14, 15). In bone marrow biopsy, reticular fibers, increased CD34+cells and more residual hematopoietic area in bone marrow biopsy specimens are helpful for the diagnosis of MDS (16, 17). Abnormal localization of immature precursor that appeared in the medullary cord can help confirm the diagnosis of MDS. In recent years, advances in the molecular pathogenesis of MDS have been greatly enriched by the systematic application of next-generation sequencing. Deep next-generation sequencing panel assays for detection of somatic mutations are now routinely available helpful in distinguishing AA from hypocellular MDS (18). Advances in novel sequencing techniques have led to the discovery of one or more gene mutations in more than 90% of all cases, and there are more than 60 genes involved (7, 8). These cytogenetic changes can help to diagnose MDS. Karyotypic abnormalities that can help doctors confirm an MDS diagnosis are present in about 50% of all cases (19). So, cytogenetic and molecular examination may not help for diagnostic value in some patients. Besides, early MDS may present no dysplasia, and the clinical symptoms are also not typical. MA and MDS both used to be classified into macrocytic anemia, and the similar manifestations of dysplasia in bone marrow increased the difficulty of differential diagnosis of the two diseases. One rapid method for assisting the diagnosis and identification of MDS, AA, and MA was very important.

Red blood cells are the most commonly and intensively studied type of blood cells in cell biology. At present, many hospitals and research institutes conducted RBCs examinations using conventional techniques which included the CBC and microscopic examinations. CBC, as the current standard technique for measuring RBCs properties, contains several important diagnostic parameters, such as the MCV, MCH, MCHC, and RBCs distribution width. Microscopic examinations classify and identify RBCs by artificially observing the morphology of cells under a microscope. Although the conventional measurement techniques can make a general view of RBCs and help identify shapes, roundness, and other information, they have difficulties in extracting high-dimensional information at the individual cell level (20). It has been challenging to establish new systems for morphological and classification analysis of erythrocytes based on the properties of individual RBCs.

In the recent years, recognition of RBCs from microscopic images using imaging processing technology has been proposed. In 2016, HA Elsalamony et al. presented algorithms capable of counting and detecting sickle and microcytic RBCs on a smear based on circular Hough transform. The neural network had been applied on their extracted data to evaluate the algorithm (21, 22). He also proposed an algorithm of assigning and counting normal, sickle RBCs and elliptocytosis (23). Other researches were conducted to screen for diseases and syndromes based on computer systems through the extraction of information of RBCs. Kim et al. (24) also demonstrated a rapid and label-free method by combining quantitative phase imaging-based single-RBC profiling with machine learning to screen for iron-deficiency anemia, reticulocytosis, hereditary spherocytosis (>98% accuracy). In Delgado-Font et al. (25) presented a high-accuracy neural network classifier for the support of sickle cell anemia by classifying RBC shape in peripheral blood images using the basic shape analysis descriptors which included circular shape factor and elliptical shape factor. The normal RBCs have a biconcave-disk shape rather than the spherical shape, with an average diameter of 7.2 μm and thickness of 2 μm. The specific shape of the RBC allows it to maximize the uptake of oxygen from its surroundings and release of carbon dioxide produced by the body. Therefore, the surface area rather than volume of the RBC is more reflective of its capacity of oxygen transportation. The above-mentioned researches have achieved some progress in extracting morphological, chemical, and mechanical properties of individual RBCs based on computer systems and even offered diagnostic support for some anemic diseases, but none of them tried to measure the area of RBCs, which may help improve the accuracy of RBC detection.

We carried out this study on the area of RBCs in peripheral blood smears of patients with MDS, AA, MA, and normal subjects by conducting RBCs segmentation and morphological analysis based on image processing technology. The results of this study showed that the mean or median RBCs area was 44.19, 42.09, 45.75, and 52.87 μm^2^, respectively in normal subjects, patients with AA, MDS, and MA. Compared with the normal subjects, the RBCs area in patients with MA was significantly higher. Compared with patients with AA and MDS, the RBCs area in patients with MA was also significantly higher. There were also significant differences between patients with AA and MDS. Therefore, we preliminarily verified the differences in the area of RBCs among patients with AA, MDS, and MA. We found no significant correlations between area of RBCs and MCV, MCH, MCHC, and RDW. Therefore, the results in this study can assist the diagnosis of patients with AA, MDS, and MA, possibly independent of standard RBC CBC indices. After comparing the diagnostic values of MCV and RBCs area in these three diseases, we found that the RBCs area showed very good diagnostic value as MCV in patients with MA. Moreover, the combined diagnostic value of MCV and RBCs area increased significantly, which could be significantly close to 1.

There are several advantages in this study. First, blood smears are easier to be obtained. Second, the area of RBCs and the differences of the area in patients with AA, MDS, and MA were preliminarily obtained. This study also has several limitations, which can be improved in the future work. First, the number of samples is not large enough. Second, cluster cells were not taken into account in this study. At last, we did not find the cutoff area values for screening patients with AA, MDS, and MA. If we get the cutoff area values to distinguish the three diseases and the methodology is mature and standardized, the detection cost and speed will be improved.

In conclusion, identifying the area of RBCs in peripheral blood smears based on the image processing technology could achieve rapid and efficient diagnostic support for patients with MDS and MA, especially for patients with MA and in combination with MCV. However, a larger sample study is needed to find the cutoff area values.

## Data Availability Statement

The raw data supporting the conclusions of this article will be made available by the authors, without undue reservation.

## Ethics Statement

The studies involving human participants were reviewed and approved by the Institutional Review Board of the Wuhan University, China. Written informed consent for participation was not required for this study in accordance with the national legislation and the institutional requirements.

## Author Contributions

YZ and XW wrote the manuscript and analyzed the results. TH and HS prepared blood smears and microscopic examination. QC processed the data. BX designed the project, provided professional guidance, and revised the manuscript. All authors contributed to the article and approved the submitted version.

## Conflict of Interest

The authors declare that the research was conducted in the absence of any commercial or financial relationships that could be construed as a potential conflict of interest.

## Publisher's Note

All claims expressed in this article are solely those of the authors and do not necessarily represent those of their affiliated organizations, or those of the publisher, the editors and the reviewers. Any product that may be evaluated in this article, or claim that may be made by its manufacturer, is not guaranteed or endorsed by the publisher.
